# Supplementary material for: SPP1 + macrophages cause exhaustion of tumor-specific T cells in liver metastases
Source: Nat Commun. 2025 May 7;16:4242. doi: 10.1038/s41467-025-59529-0 (PMC12059142; doi:10.1038/s41467-025-59529-0)
Supplement: Supplementary file 1 — Supplementary Information [file 41467_2025_59529_MOESM1_ESM.pdf]

Supplementary Figure 1. Organ Specific TAS CD8+ T cell Response. Absolute counts of AH1+ CD8+ T cells at day 14 (n=5; IH TIL vs IH Liver p= 0.0008; IH TIL vs SQ Liver p= 0.0005; IH TIL vs SQ TIL p= 0.0192; IH TIL vs TF Liver p= 0.0005) (A), and at day 21 in the liver (IH, n=7; SQ, n=5; IH Liver vs SQ Liver p= 0.0053; IH Liver vs TF Liver p= 0.0038) (B) and TILs (IH, n=7; SQ, n=5) (C) in CT26 tumor-bearing female, BALB/c 6-8 week old mice. Frequency of AH1+ CD8+ T cells in liver (n=4) (D), pulmonary (n=5; Liver vs Hilar LN p<0.0001; Lung vs. Hilar LN p=0.0005; Spleen vs. Hilar LN p=0.0002; Cutenous LN vs. Hilar LN p=0.0005; Hilar LN vs. Portal LN p=0.0001) (E), and subcutaneous (n=H) (F) CT26 tumor-bearing female, BALB/c 6-8 week old mice. Frequency of AH1+ CD8+ T cells in liver (n=5; Liver vs. TIL p<0.0001; Lung vs. TIL p<0.0001; Spleen vs. TIL p<0.0001; Cutenous LN vs. TIL p<0.0001; Hilar LN vs. TIL p<0.0001; Portal LN vs. TIL p<0.0001) (G), pulmonary (n=5; Liver vs. Lung p=0.0018; Lung vs. Cutenous LN p=0.0073; Lung vs. Hilar LN p=0.0005; Lung vs. Portal LN p=0.0003) (H), and subcutaneous (n=6; Liver vs. TIL p<0.0001; Lung vs. TIL p<0.0001; Spleen vs. TIL p<0.0001; Cutenous LN vs. TIL p<0.0001; Hilar LN vs. TIL p<0.0001; Portal LN vs. TIL p<0.0001) (I) B16F10 tumor-bearing female C57BL/6 6-8 week old mice. Data was analyzed by ordinary one-way ANOVA (B-C, F-H,J-L): *P < 0.05; **P < 0.01; ***P < 0.005; ****P < 0.001, ns = not significant (data are presented as mean values +/- SEM).

Supplementary Figure 2. Flow gating strategy of TAS CD8+ T cells. Flow gating strategy for Figures 1, 2, 5, 7 and Supplementary Figures 1 and 3.

Supplementary Figure 3. Further insights into the TAS CD8+ T cell phenotype and function. Frequency of A) TIM-3+ and C) PD-1+ among TAS CD8+ T cells in the TIL, liver and spleen of liver and subcutaneous tumor-bearing mice at days 7 (subcutaneous (SQ), n=5; intrahepatic (IH) tumor injection, n=5), 14 (SQ, n=5; IH, n=5) and 21(SQ, n=5; IH, n=7). Comparison of B) TIM-3+ (Liver p= 0.000034; TIL p=0.000034) and D) PD-1+ (Liver p= 0.000006; TIL p=0.000501) AH1+ to AH1- CD8+ T cells in the liver (left, n=17) and TIL (right, n=17) at day 14. E) Flow cytometry staining of CD8+ T cells after CD8+ T cell depletion (IH, n=7;IgG,n=11) or IgG treatment (IH, n=7;IgG,n=10) (IH IgG vs aCD8 p<0.0001; SQ IgG vs aCD8 p<0.0001). F) Tumor size measurements after CD8+ T cell depletion (n=11) compared to IgG control (n=10) after subcutaneous injection (day 9 p=0.0007; day 12 p<0.0001). All experiments (A-F) were completed in female, BALB/c 6-8 week old mice. Data was analyzed with unpaired t-tests (B and D), ordinary one-way ANOVA (E) and unpaired t-test (F): *P < 0.05; **P < 0.01; ***P < 0.005; ****P < 0.001, ns = not significant (data are presented as mean values +/- SEM).

Supplementary Figure 4. UMAP of CD8+ T cell clusters and pTRT scores. UMAP showing (A) all cells (n=10 patients), (B) myeloid clusters (n=10 patients), (C) CD8+ T cell clusters and pTRT score based on scGSEA analysis (n=10 patients) as a (D) feature plot (n=10 patients), (E) ridge plot per cluster (n=10 patients), and (F) ridge plot per patient (n=10 patients).

Supplementary Figure 5. pTRT in human single cell analysis. A) scGSEA analysis using same methodology as Figure 3A except with a random set of 500 genes (n=10). B) pTRT frequency of CD8+ T cells in hepatic metastasis compared to primary CRC shown as paired analysis (n=10; p=0.0426). Data was analyzed by one-way ANOVA (A) and paired t-test (B):

*P < 0.05; **P < 0.01; ***P < 0.005; ****P < 0.001, ns = not significant (data are presented as mean values +/- SEM).

Supplementary Figure 6. CellChat analysis of metastatic tumor, surrounding hepatic tissue and surrounding colonic tissue. A) CellChat cellular communication analysis for communications that the pTRT are sending to all major cell types comparing the strength of interactions between hepatic metastasis (MT) to surrounding hepatic tissue (MN). B) CellChat cellular communication analysis for communications that the pTRT are receiving from all major cell types comparing hepatic metastasis to surrounding hepatic tissue. C) CellChat cellular communication analysis for communications that the pTRT are sending to all major cell types comparing the strength of interactions between surrounding hepatic tissue (MN) to surrounding colonic tissue (PN). D) CellChat cellular communication analysis for communications that the pTRT are receiving from all major cell types comparing the strength of interactions between surrounding hepatic tissue (MN) to surrounding colonic tissue (PN). Data was analyzed paired wilcox test (default for CellChat) (A-D).

Supplementary Figure 7. Flow gating strategy of macrophages. Flow gating strategy for Figure 4 and Supplementary Figure 8 in female, BALB/c 6-8 week old mice.

Supplementary Figure 8. **SPP1 macrophage isolation.** A) Peritoneal lymphocytes prior to sorting for F4/80+ macrophages (n=1 mouse). B) Peritoneal lymphocytes after sorting for F4/80+ macrophages (n=1 mouse). C) qPCR analysis of SPP1 in sorted macrophages in normoxic or hypoxic conditions (n=1 mouse). D) Splenic lymphocytes before and after sorting for CD8+ T cells (n=1 mouse). Experiments were completed in female, BALB/c 6-8 week old mice (A-C) and female, C57BL/6 6-8 week old mice (D).

Supplementary Figure 9. M2 Correlation in the setting of profibrotic polarization in the liver. **A)** SPP1 CellChat pathway analysis for communication to pTRT in HCC scRNA-seq data (n=15). Correlation of fibronectin expression in metastatic intrahepatic tumors (MT) to TLR-2 (B) and TLR-4 (C) in primary tumors (n=10). Correlation of fibronectin expression in primary CRC tumors (PT) to TLR-2 (D) and TLR-4 (E) in primary tumors (n=10). Correlation of SPP1 expression by macrophages in metastatic intrahepatic tumors (MT) to TLR-2 (F) and TLR-4 (G) expression in primary tumors (n=10). Correlation of SPP1 expression by macrophages in primary CRC tumors to TLR-2 (H) and TLR-4 (I) in primary tumors (n=10). Correlation of metastatic intrahepatic tumor (MT) CD163 expression by macrophages to primary tumor TGF-β (J), IL-6 (K), TLR-2 (L), TLR-4 (M) (n=10). Correlation of metastatic intrahepatic tumor (MT) CD206 expression by macrophages to primary tumor TGF-β (N), IL-6 (O), TLR-2 (P), TLR-4 (Q) (n=10). Data was analyzed using and simple linear regression (B-Q) (data are presented as mean values +/- SEM).

Supplementary Figure 10. Pseudotime, scRNA seq and Bulk Analysis of VSIG4 SPP1 expressing macrophages. VSIG4 and CSF1R feature plot of intrahepatic macrophage and monocyte population from metastatic CRC tumor-bearing liver (A) (n=10). VSIG4 and CSF1R feature plot of intracolonic macrophage and monocyte population (n=10) (B). Top genes in rank order correlating with liver pseudotime trajectory analysis (n=10) (C) with SPP1 boxed.

Pseudotime trajectory analysis showing correlation of collagen regulation score (GO:0032967; positive regulation of collagen biosynthetic process) (D) with pseudotime trajectories in the liver (n=10). Intracolonic macrophage and monocyte population UMAP (n=10) from primary CRC tumor-bearing colon (E). Pseudotime trajectory analysis (n=10) of intracolonic macrophages and monocytes (F). Top genes correlating with colonic pseudotime trajectory analysis (n=10) (C). Pseudotime trajectory analysis showing correlation of SPP1 (H), TGF-βR1 and IL6R (I), CSF1R and VSIG4 (J), and CD163 and CD206 (K) with pseudotime trajectories in the colon (n=10). Bulk TCGA survival analysis of HCC (left; n=438) and CRC (right; n=551) based on gene signature expression (L). UMAP (left) as well as feature plot (middle) and violin plot (right) of CD68 expression per cluster where cluster 2 and 3 were defined as CD68+ (M). Kupfer cell and SPP1 marker expression per clinical group (N). Cell-type icons created with BioRender.com (A-C).[89] Data was analyzed using Morans I (C and D, G-K) and p-value (chi-squared distribution) (L) (data are presented as mean values +/- SEM).

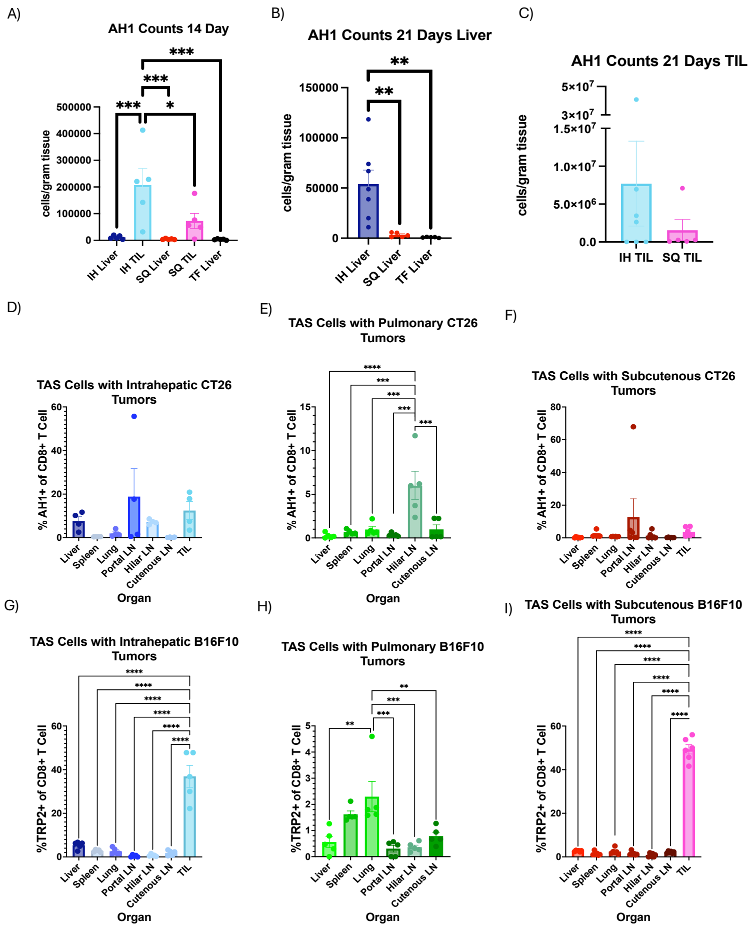

Supplementary Figure 1.

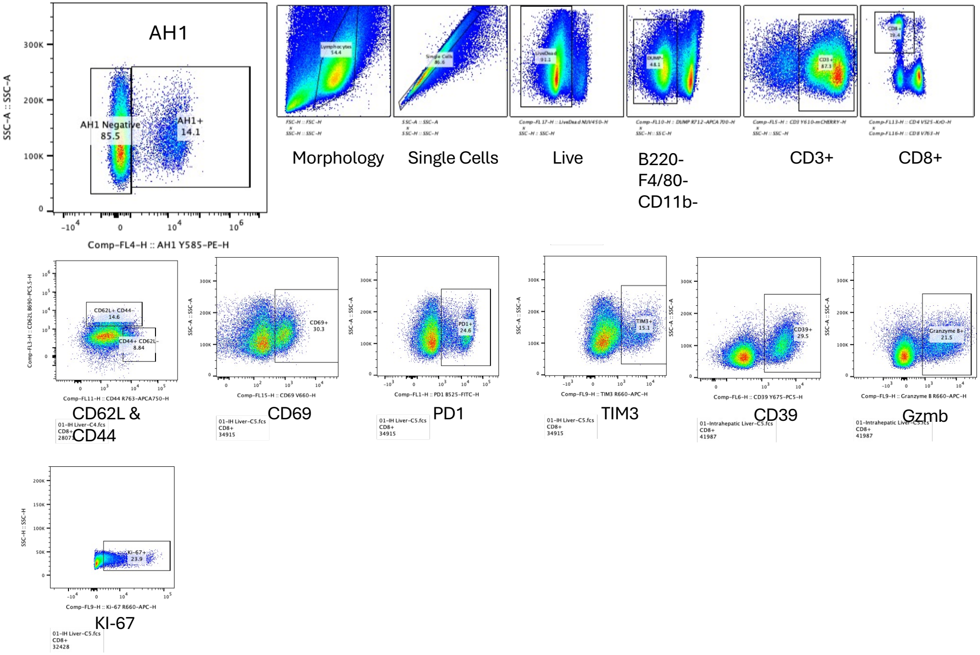

Supplementary Figure 2.

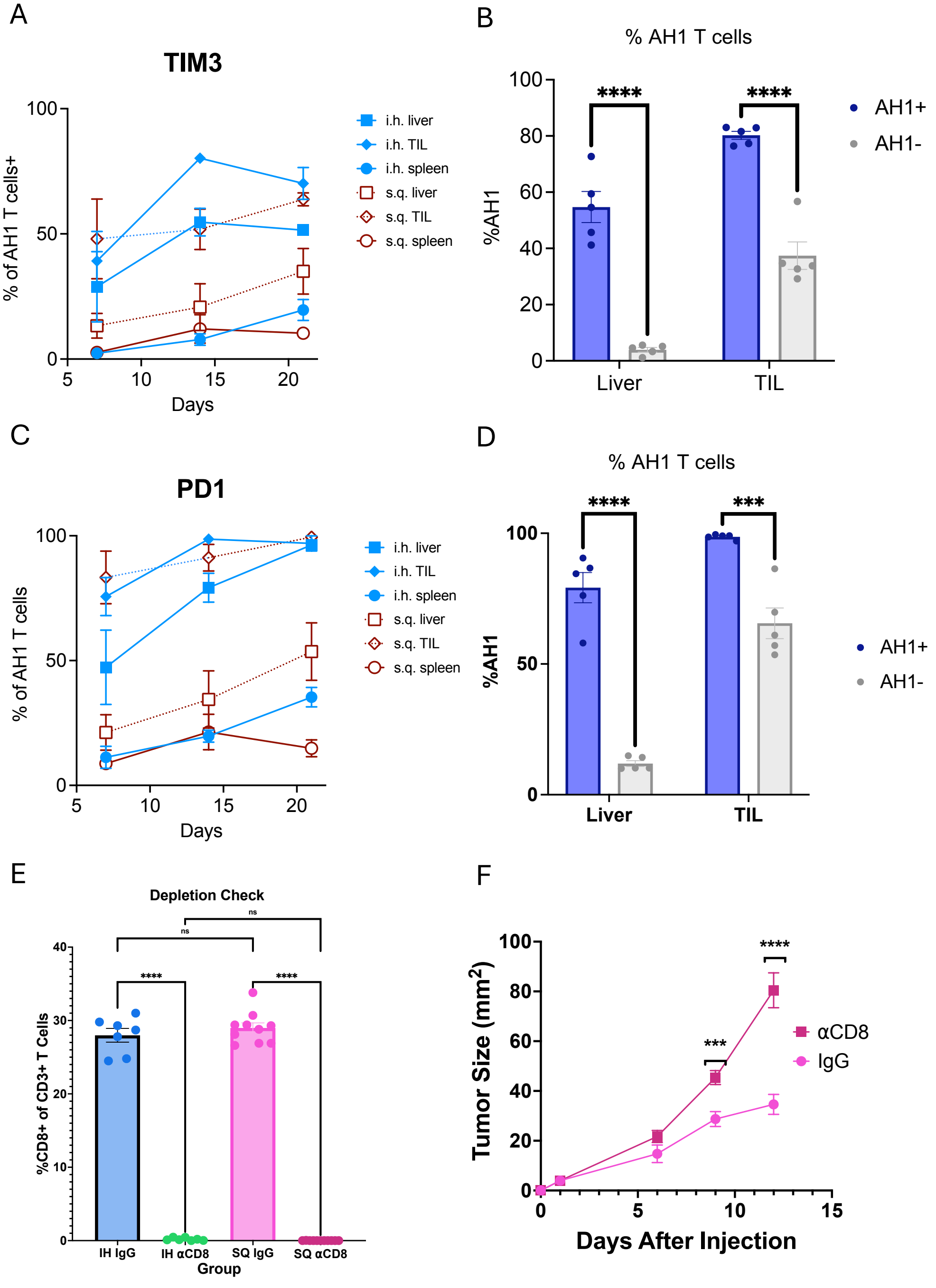

Supplementary Figure 3.

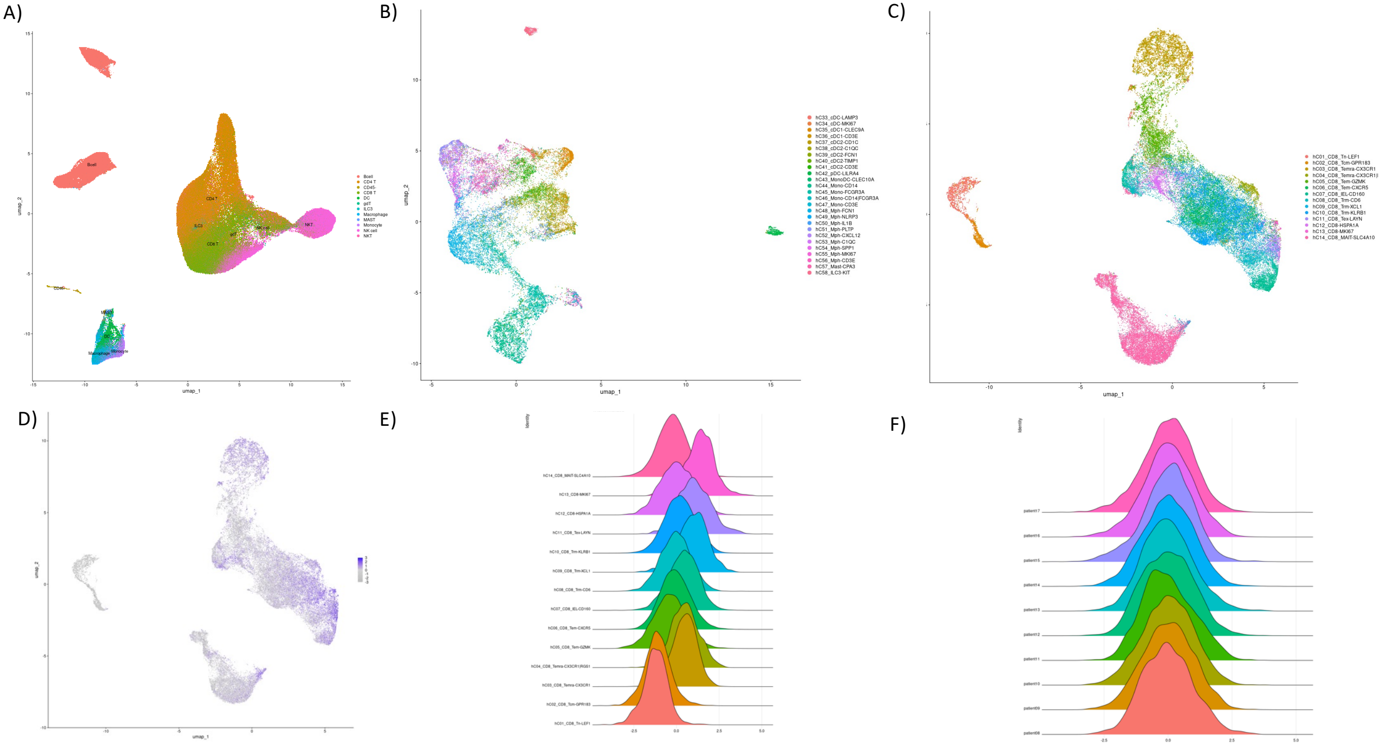

Supplementary Figure 4.

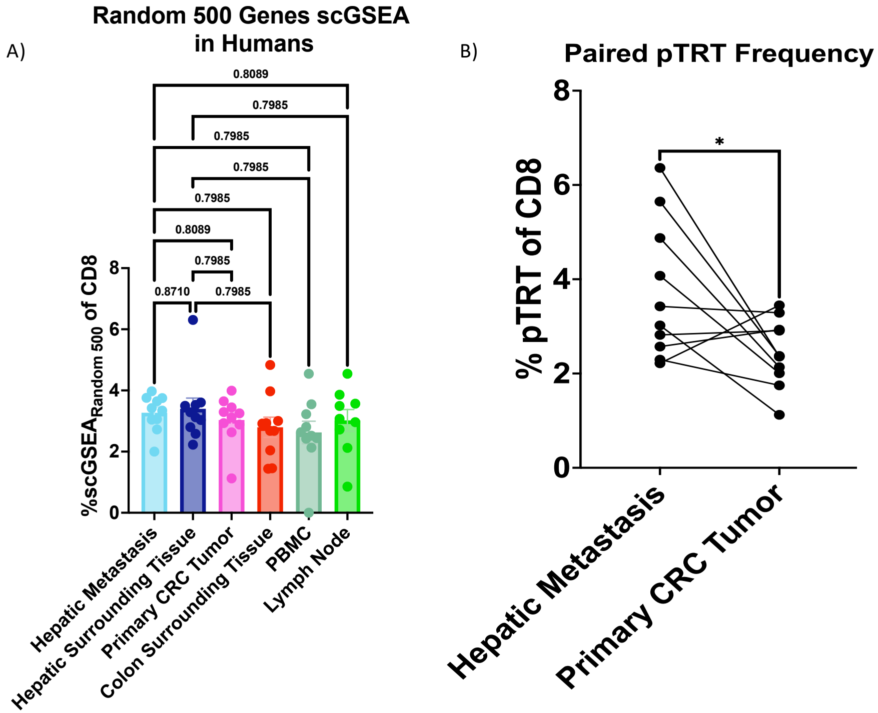

Supplementary Figure 5.

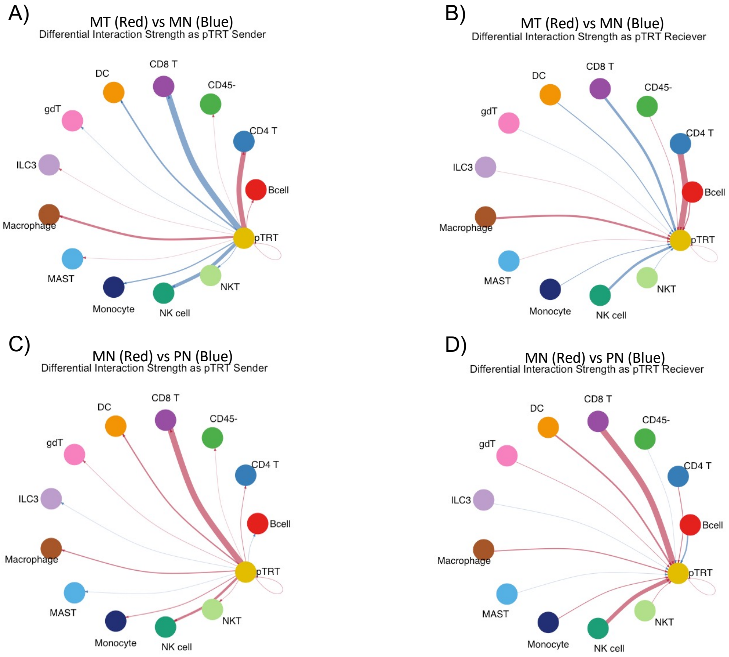

Supplementary Figure 6.

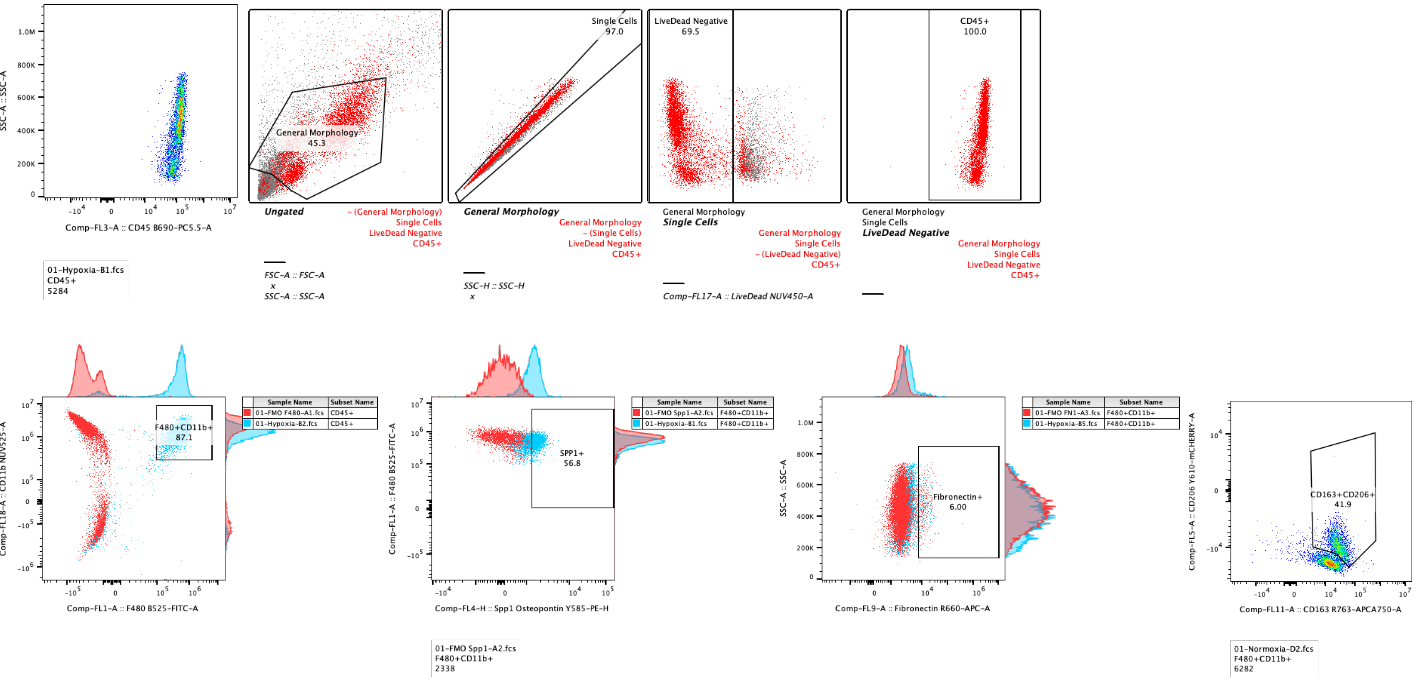

Supplementary Figure 7.

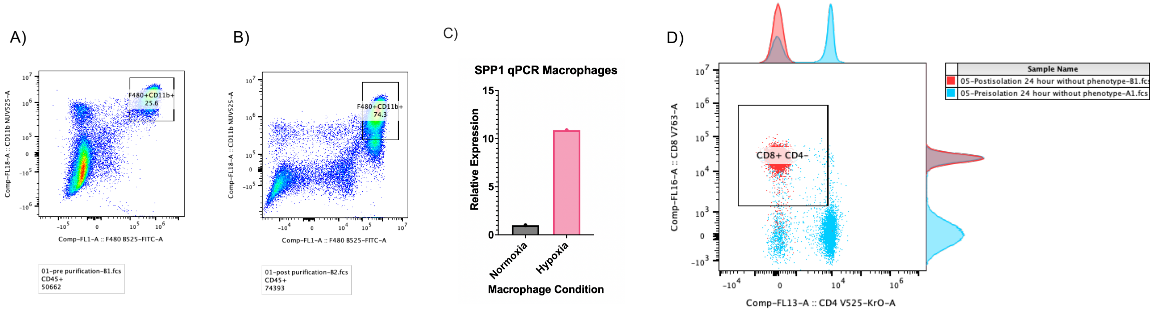

Supplementary Figure 8. SPP1 macrophage and CD8 T cell isolation.

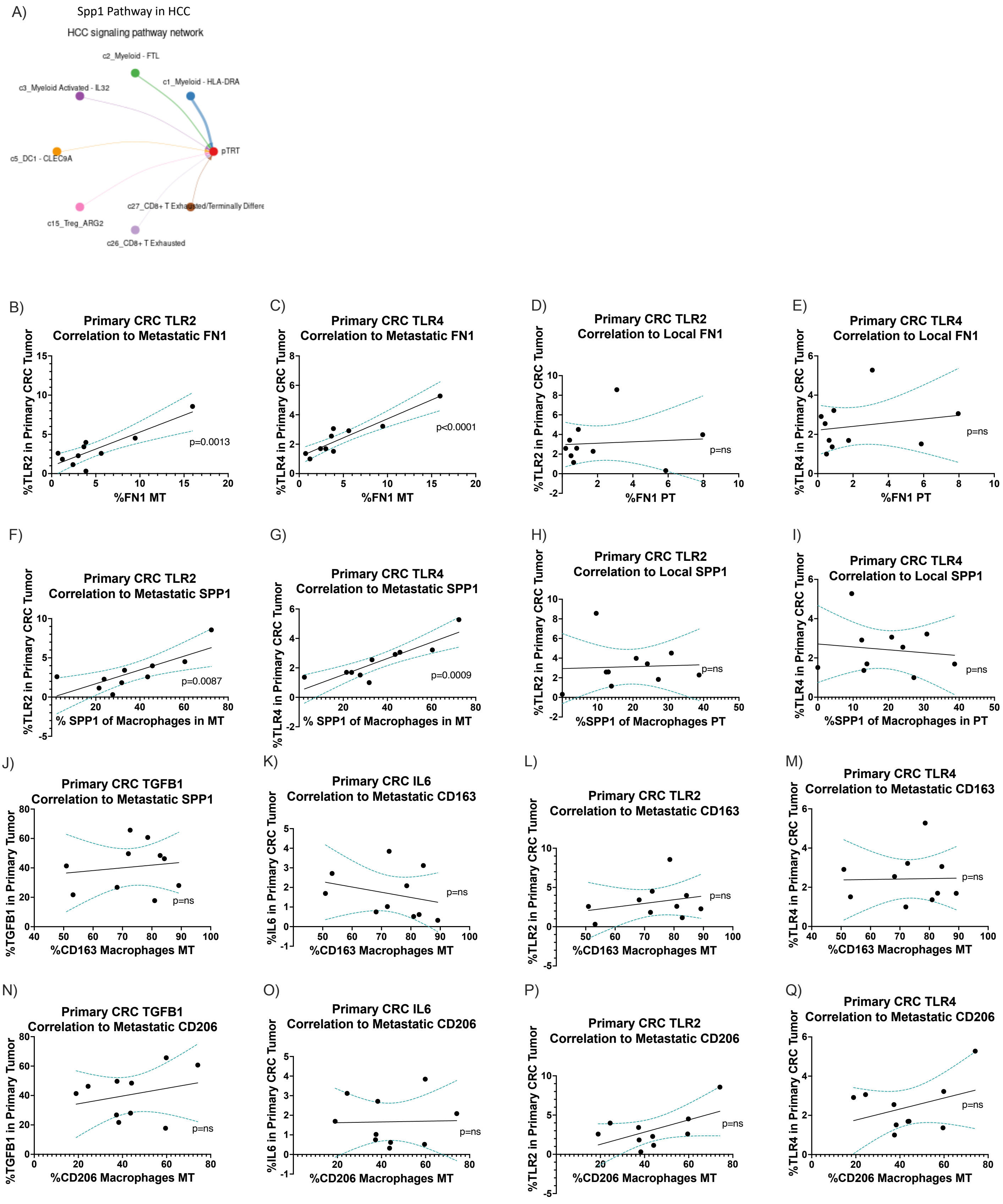

Supplementary Figure 9.

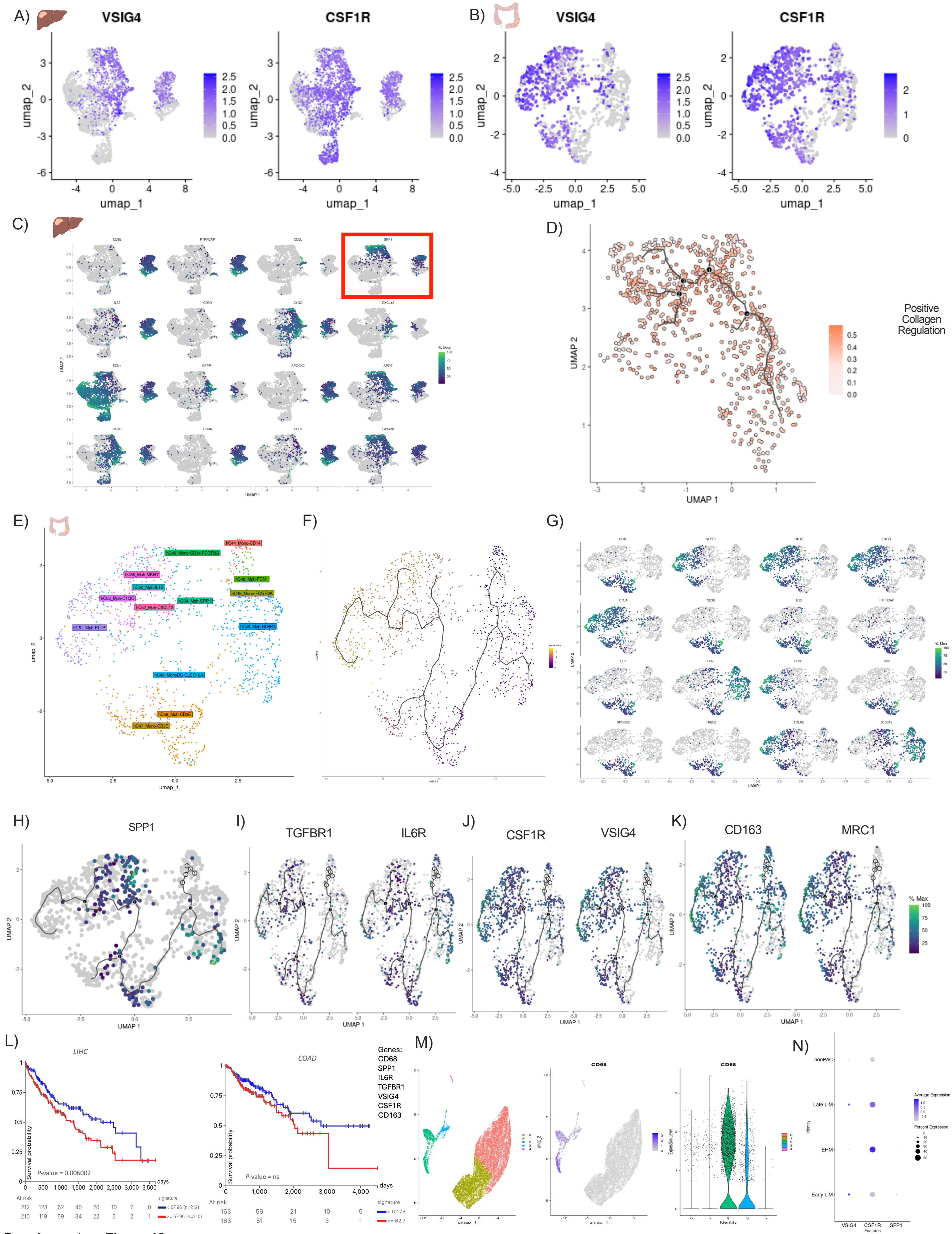

Supplementary Figure 10.
