# Supplementary material for: SPP1 + macrophages cause exhaustion of tumor-specific T cells in liver metastases
Source: Nat Commun. 2025 May 7;16:4242. doi: 10.1038/s41467-025-59529-0 (PMC12059142; doi:10.1038/s41467-025-59529-0)
Supplement: Supplementary file 2 — Reporting Summary [file 41467_2025_59529_MOESM2_ESM.pdf]

nature portfolio

Corresponding author(s): Tim Greten

Last updated by author(s): Apr 15, 2025

Reporting Summary

Nature Portfolio wishes to improve the reproducibility of the work that we publish. This form provides structure for consistency and transparency in reporting. For further information on Nature Portfolio policies, see our Editorial Policies and the Editorial Policy Checklist.

Statistics

For all statistical analyses, confirm that the following items are present in the figure legend, table legend, main text, or Methods section.

n/a	Confirmed	
☐	☒	The exact sample size (*n*) for each experimental group/condition, given as a discrete number and unit of measurement
☐	☒	A statement on whether measurements were taken from distinct samples or whether the same sample was measured repeatedly
☐	☒	The statistical test(s) used AND whether they are one- or two-sided<br>*Only common tests should be described solely by name; describe more complex techniques in the Methods section.*
☐	☒	A description of all covariates tested
☐	☒	A description of any assumptions or corrections, such as tests of normality and adjustment for multiple comparisons
☐	☒	A full description of the statistical parameters including central tendency (e.g. means) or other basic estimates (e.g. regression coefficient) AND variation (e.g. standard deviation) or associated estimates of uncertainty (e.g. confidence intervals)
☐	☒	For null hypothesis testing, the test statistic (e.g. *F*, *t*, *r*) with confidence intervals, effect sizes, degrees of freedom and *P* value noted<br>*Give P values as exact values whenever suitable.*
☒	☐	For Bayesian analysis, information on the choice of priors and Markov chain Monte Carlo settings
☐	☒	For hierarchical and complex designs, identification of the appropriate level for tests and full reporting of outcomes
☒	☐	Estimates of effect sizes (e.g. Cohen's *d*, Pearson's *r*), indicating how they were calculated

Our web collection on statistics for biologists contains articles on many of the points above.

Software and code

Policy information about availability of computer code

Data collection	For HCC scRNAseq and CODEX Data:<br>1. The human single-cell sequencing data are deposited through dbGaP (accession number: phs003279.v1.p1):<br>https://www.ncbi.nlm.nih.gov/projects/gap/cgi-bin/study.cgi?study_id=phs003279.v1.p1<br>2. The raw CODEX images are hosted at The Cancer Imaging Archives (TCIA) under<br>https://doi.org/10.7937/bh0r-y074.<br>Data was collected from cited sources.<br><br>scRNAseq of CRC Metastasis:<br>The processed gene expression data of this study can be obtained from Gene Expression Omnibus (GEO) with an accession number of<br>GSE164522<br>. Analysis and visualization of scRNA-seq datasets can be performed at<br>http://cancer-pku.cn:3838/CRLM/<br>(username: CRLM, password: CRLM).<br><br>scRNAseq of pancreatic adenocarcinoma:<br>Gene expression data have been deposited to the National Institutes of Health Gene Expression Omnibus repository and can be accessed as GSE245535 (bulk mRNA-seq) and GSE267209 (scRNA-seq).
Data analysis	GraphPad Prism 9 and 10 (GraphPad Prism, RRID:SCR_002798) was utilized for statistical and visual output. Human data analyses were performed using R (vs. 4.2.1) on RStudio (vs. 2022.070.01). No custom code was required and all relevant citations to previously produced

code is found in the manuscript. Seurat analysis was completed using Seurat package (vs 4.3.0.1) under Rstudio (vs 2023.09.1+494). Monocole and Pseudotime analysis were completed using the Monocle3 package (version 1.3.4).The nSolver 4.0 software was used to normalize expression values using housekeeping genes following the manufacturer's recommendations. For Codex, analysis, HALO Image analysis software (CODEX processor version 1.7.0.6 without segmentation and HALO image analysis platform V3.2, Indica Labs available through the NCI HALO Image Analysis Resource).

For manuscripts utilizing custom algorithms or software that are central to the research but not yet described in published literature, software must be made available to editors and reviewers. We strongly encourage code deposition in a community repository (e.g. GitHub). See the Nature Portfolio guidelines for submitting code & software for further information.

Data

Policy information about availability of data

All manuscripts must include a data availability statement. This statement should provide the following information, where applicable:

- Accession codes, unique identifiers, or web links for publicly available datasets
- A description of any restrictions on data availability
- For clinical datasets or third party data, please ensure that the statement adheres to our policy

The human single-cell sequencing data from HCC patients are deposited through dbGaP (accession number: phs003279.v1.p1): https://www. ncbi.nlm.nih.gov/projects/gap/cgi-bin/study.cgi?study_id=phs003279.v1.p1 (HCC)70. The raw CODEX images are hosted at The Cancer Imaging Archives (TCIA) under https://doi.org/10.7937/bh0r-y074. Regarding publicly available datasets not generated by our lab, the following accession numbers provide access to both raw and processed data: GSE164522 (primary CRC and corresponding liver metastasis; https://www.ncbi.nlm.nih.gov/geo/query/acc.cgi?acc=GSE164522)24, GSE245535 (bulk mRNA-seq; https://www.ncbi.nlm.nih.gov/geo/query/acc.cgi?acc=GSE245535)48 and GSE267209 (pancreatic cancer and corresponding metastasis; https://www.ncbi.nlm.nih.gov/geo/query/acc.cgi?acc=GSE267209)48. Source data are provided with this paper.

Research involving human participants, their data, or biological material

Policy information about studies with human participants or human data. See also policy information about sex, gender (identity/presentation), and sexual orientation and race, ethnicity and racism.

Reporting on sex and gender	N/a
Reporting on race, ethnicity, or other socially relevant groupings	N/a
Population characteristics	N/a
Recruitment	N/a
Ethics oversight	N/a

Note that full information on the approval of the study protocol must also be provided in the manuscript.

Field-specific reporting

Please select the one below that is the best fit for your research. If you are not sure, read the appropriate sections before making your selection.

[x] Life sciences [ ] Behavioural & social sciences [ ] Ecological, evolutionary & environmental sciences

For a reference copy of the document with all sections, see nature.com/documents/nr-reporting-summary-flat.pdf

Life sciences study design

All studies must disclose on these points even when the disclosure is negative.

Sample size	Sample sizes can all be found in figure legends. Sample sizes for animal studies were guided by previous studies with similar or identical tumor models (no statistical method was used to predetermine sample size). Citation:<br>Ma, C. et al. Gut microbiome–mediated bile acid metabolism regulates liver cancer via NKT cells. Science 360, eaan5931 (2018).<br>69. Ma, C. et al. Platelets control liver tumor growth through P2Y12-dependent CD40L release in NAFLD. Cancer Cell 40, 986-998.e5 (2022).<br>70. Ruf, B. et al. Tumor-associated macrophages trigger MAIT cell dysfunction at the HCC invasive margin. Cell 186, 3686-3705.e32 (2023).<br>Huang, P. et al. Hepatic immune environment differences among common mouse strains in models of MASH and liver cancer. JHEPReport 0, (2025).<br>86. Myojin, Y. et al. Multiomics analysis of immune correlatives in hepatocellular carcinoma patients treated with tremelimumab plus durvalumab. Gut (2025) doi:10.1136/gutjnl-2024-334026.<br>87. Benmebarek, M.-R. et al. Anti-vascular endothelial growth factor treatment potentiates immune checkpoint blockade through a BAFF- and IL-12-dependent reprogramming of the TME. Immunity 58, 926-945.e10 (2025).
Data exclusions	No data was excluded from study.
Replication	All attempts of replication were successful including for CD8 depletion tumor sizes and SPP1 KO. The majority of experiments were repeated at least twice to obtain robust data for indicated statistical analyses.

Randomization	Mice were randomly allocated to cages and groups.
Blinding	All tumor weights and sizes were measured by a blinded author.

Reporting for specific materials, systems and methods

We require information from authors about some types of materials, experimental systems and methods used in many studies. Here, indicate whether each material, system or method listed is relevant to your study. If you are not sure if a list item applies to your research, read the appropriate section before selecting a response.

Materials & experimental systems

n/a	Involved in the study
☐	☒ Antibodies
☐	☒ Eukaryotic cell lines
☒	☐ Palaeontology and archaeology
☐	☒ Animals and other organisms
☒	☐ Clinical data
☒	☐ Dual use research of concern
☒	☐ Plants

Methods

n/a	Involved in the study
☒	☐ ChIP-seq
☐	☒ Flow cytometry
☒	☐ MRI-based neuroimaging

Antibodies

Antibodies used	The following antibodies were used (dilution for each per manufacture instructions) for flow cytometry analysis: anti-CD62L-PerCP/Cy5.5 (BioLegend, Cat #104432, Clone: MEL-14, dilution 1:200), anti-CD44-APC/Cy7 (BioLegend, Cat #103028, Clone: IM7, dilution 1:200), anti-CD69-BV650 (BioLegend, Cat #104541, Clone:H1.2F3, dilution 1:100), anti-NK1.1-BV510 (BioLegend, Cat #108738, Clone: PK136, dilution 1:200), anti-B220-Alexa Fluor 700 (BioLegend, Cat #103232, Clone: RA3-6B2, dilution 1:400), anti-CD3-Alexa Fluor 594 (BioLegend, Cat #100240, Clone: 17A2, dilution 1:200), anti-CD3-Alexa Fluor 700 (BioLegend, Cat #100216, Clone: 17A2, dilution 1:200), anti-CD4-BV605 (BioLegend, Cat #100451, Clone: GK1.5, dilution 1:200), anti-CD4-BV510 (BioLegend, Cat #100449, Clone: GK1.5, dilution 1:200), anti-CD8-BV786 (BD Biosciences, Cat #563332, Clone: 53-6.7, dilution 1:200), anti-CD39-APC (BioLegend, Cat #143809, Clone: Duha59, dilution 1:100), anti-TIM-3-BV421 (BioLegend, Cat #119723, Clone: RMT3-23, dilution 1:100), anti-TIM-3-BV605 (BioLegend, Cat #119721, Clone: RMT3-23, dilution 1:100), anti-TIM-3-APC (BioLegend, Cat #119706, Clone: RMT3-23, dilution 1:100), anti-PD-1-FITC (BioLegend, Cat #135214, Clone: 29F.1A12, dilution 1:100), anti-Ly-6G-BV650 (BioLegend, Cat #127641, Clone: 1A8, dilution 1:100), anti-Ly-6C-FITC (BioLegend, Cat #128006, Clone: HK1.4, dilution 1:100), anti-CD19-PErCp-Cy5.5 (BioLegend, Cat #152406, Clone: 1D3/CD19, dilution 1:200), anti-CD25-PerCp-Cy5.5 (BioLegend, Cat #101911, Clone: 3C7, dilution 1:200), anti-TCR-b -PE/Cy7 (BioLegend, Cat #109222, Clone: H57-597, dilution 1:300), anti-LAG-3-BUV496 (BD Biosciences, Cat #750027, Clone: C9B7W, dilution 1:100), anti-CD45-PerCp-Cy5.5 (BioLegend, Cat #157208, Clone: S18009F, dilution 1:100), anti-F4/80-Alexa Fluor 700 (BioLegend, Cat# 123130, Clone: BM8; dilution 1:400), anti-F4/80-FITC (BioLegend, Cat #123108, Clone: BM8, dilution 1:100), anti-F4/80-APC (Invitrogen, Cat #17-4801-80, Clone: BM8), anti-F4/80-PE (BioLegend, Cat #111704, Clone: W20065D, dilution 1:100) anti-CD11b-Alexa Fluor 700 (BioLegend, Cat #101222, Clone: M1/70, dilution 1:400), anti-CD11b-APC/Cy7 (BioLegend, Cat #101226, Clone: M1/70, dilution 1:200), anti-CD11b- BUV496 (BD Biosciences, Cat #749864, Clone: M1/70, dilution 1:200), anti-CD68- PerCp-Cy5.5 (BioLegend, Cat #137010, Clone: FA-11, dilution 1:200), anti-CD80-BV650 (BioLegend, Cat #104732, Clone: 16-10A1, dilution 1:100), anti-CD86-BV605 (BioLegend, Cat #105037, Clone: GL-1, dilution 1:100), anti-CD163-APC/Cy7 (BioLegend, Cat #155324, Clone: S150491, dilution 1:100), anti-CD206-AF594 (BioLegend, Cat #141726, Clone: C068C2, dilution 1:100), anti-CD274(PD-L1)-BV786 (BD Biosciences, Cat #741014, Clone: MIH5, dilution 1:100), and anti-CD183(CXCR3)-BV510 (BioLegend, Cat #126527, Clone: CXCR3-173, dilution 1:100). The following tetramers (dilution 1:200), from the NIH tetramer core facility in collaboration with Emory University, were used: OT-1 tetramer on APC, OT-1 tetramer on PE, AH1 tetramer on APC, AH1 tetramer on PE, TRP2 tetramer on APC, and TRP2 tetramer on PE. The following antibodies were used for intracellular cytokine and transcription factor staining using the BD Cytofix/Cytoperm™ Fixation/Permeabilization Kit (BD Biosciences, Cat #554714) or the BD Pharmingen™ Transcription Factor Buffer Set (BD Biosciences, Cat #562574) following the manufacturer's recommendation: anti-Granzyme B-APC (BioLegend, Cat# 396408, Clone: QA18A28, dilution 1:100), anti-Granzyme B-FITC (BioLegend, Cat #515403, Clone: GB11, dilution 1:100), anti-IFN-γ-BV650 (BioLegend, Cat #505832, Clone: XMG1.2, dilution 1:100), anti-OPN-PE (R&D Systems, Cat #IC808P, dilution 1:100), Osteopontin Rabbit PolyAb-CoraLite Plus 488 (Proteintech, Cat#CL48822952100UL, dilution 1:100), anti-CXCL9-APC (BioLegend, Cat #515606, Clone: MIG-2F5.5, dilution 1:100), anti-Fibronectin-1 Alexa Fluor 647 (BD biosciences, Cat #563098, Clone: 10/Fibronectin, dilution 1:200), anti-FoxP3-BV421 (BioLegend, Cat #126419, Clone: MF-14, dilution 1:100), anti- FoxP3-PerCp-Cy5.5 (BD Biosciences, Cat #563902, Clone: R16-715, dilution 1:100), anti- Ki-67-BV421(BioLegend, Cat #652411, Clone: 16A8, dilution 1:100), anti- Ki-67-BV421 (Invitrogen, Ref #364-5698-82, dilution 1:100), and anti-Ki-67-APC (BioLegend, Cat #652405, Clone: 16A8, dilution 1:100). Samples were run on a CytoFLEX LX flow cytometer (Beckman Coulter CytoFLEX Flow Cytometer, RRID: SCR_019627) and data was analyzed using FlowJo software (FlowJo, RRID:SCR_008520) software.Depletion of CD8+ T cells were conducted by 100 µL intraperitoneal injection containing 10 µg of antibody per gram of mouse weight (InVivoMAb anti-mouse CD8α, CAT# BE0061, Clone 2.43, BioxCell). Isotype-matched IgG2b (InVivoMAb rat IgG2b isotype control, anti-keyhole limpet hemocyanin, CAT# BE0090,Clone LTF-2, BioxCell) was administered at 10 ug antibody/g mouse to IgG controls.
Validation	Validation can be found in Supplemental Figure 3.

Eukaryotic cell lines

Policy information about cell lines and Sex and Gender in Research

Cell line source(s)	The B16-F10 melanoma cell line was a gift from Ugur Sahin (Mainz, Germany; ATCC CRL-6475 lot no. 58078645). There is no record of the sex of the originating mouse.<br>CT26 was obtained was obtained from ATCC (ATCC, catalog no. CRL-2638, RRID:CVCL_7256). We believe based on the ATCC website that these were made from female mice.<br>The RIL-175 were generated originally described in Kapanadze et al., 2013 in our lab. There is no record of sex of the originating mouse.
Authentication	The cell lines used in this article were not authenticated in the past year.
Mycoplasma contamination	All cell lines tested negative for Mycoplasma.
Commonly misidentified lines (See ICLAC register)	*Name any commonly misidentified cell lines used in the study and provide a rationale for their use.*

Animals and other research organisms

Policy information about studies involving animals; ARRIVE guidelines recommended for reporting animal research, and Sex and Gender in Research

Laboratory animals	Two- to four-month-old female BALB/c mice (strain code #028) were purchased from Charles River Laboratories. Two- to four-month-old female B6(Cg)-Tyrc-2J/J (strain code #000058) were purchased from The Jackson Laboratory. Two- to four-month-old female and male C57BL/6J mice (strain code #000664) were purchased from The Jackson Laboratory. Two- to four-month-old female and male B6.129S6(Cg)-Spp1tm1Blh/J (OPN KO) (strain code #004936; RRID:IMSR_JAX:004936) were purchased from The Jackson Laboratory.
Wild animals	No wild animals were used.
Reporting on sex	Sex was reported in figure legends for relevant, key experiments throughout the study. For key validation experiments, a mixed used of male and female mice was used to ensure that the results applied to both sexes.
Field-collected samples	No field collected samples were used.
Ethics oversight	Regarding ethical approval, wll technical procedures and experimental endpoints were detailed in the animal study proposals (MOB-15, MOB-23, MOB-17, MOB-28) which were approved by the NCI Division of Intramural Research Animal Care and Use Committee. The mice were kept in the CRC animal facility at NCI, following the guidelines outlined in the National Research Council Guide for the Care and Use of Laboratory Animals.

Note that full information on the approval of the study protocol must also be provided in the manuscript.

Plants

Seed stocks	*Report on the source of all seed stocks or other plant material used. If applicable, state the seed stock centre and catalogue number. If plant specimens were collected from the field, describe the collection location, date and sampling procedures.*
Novel plant genotypes	*Describe the methods by which all novel plant genotypes were produced. This includes those generated by transgenic approaches, gene editing, chemical/radiation-based mutagenesis and hybridization. For transgenic lines, describe the transformation method, the number of independent lines analyzed and the generation upon which experiments were performed. For gene-edited lines, describe the editor used, the endogenous sequence targeted for editing, the targeting guide RNA sequence (if applicable) and how the editor was applied.*
Authentication	*Describe any authentication procedures for each seed stock used or novel genotype generated. Describe any experiments used to assess the effect of a mutation and, where applicable, how potential secondary effects (e.g. second site T-DNA insertions, mosiacism, off-target gene editing) were examined.*

Flow Cytometry

Plots

Confirm that:

- [x] The axis labels state the marker and fluorochrome used (e.g. CD4-FITC).
- [x] The axis scales are clearly visible. Include numbers along axes only for bottom left plot of group (a 'group' is an analysis of identical markers).
- [x] All plots are contour plots with outliers or pseudocolor plots.
- [x] A numerical value for number of cells or percentage (with statistics) is provided.

Methodology

Sample preparation	From liver, spleen, and tumor, single cells were isolated, as previously well described.4 Briefly, the organs of the euthanized mice were removed. The tumors from tumor-bearing livers were separated from the liver, to be processed independently, as well as tumors from subcutaneous tissue. For CT26 tumors, tumor tissue was digested for 45 minutes at 37C at 220 RPM using RPMI 1640 with 1mg/mL Collagenase D (Sigma-Aldrich, Cat #C5138) and 50 unit/mL DNase I (STEMCELL Technologies, Cat #07900) where digestion was halted using 10% FCS PBS. For B16F10 tumor tissue, tumor tissue was homogenized through mechanical digestion and filtered through 70 µm nylon mesh. Tumors then underwent density-gradient centrifugation with Lympholyte (Cedarlane, Cat #CL5035) at 850g at room temperature (acceleration 9, deacceleration 0) where the middle layer was kept for further analysis. Of note, Lympholyte was carefully layered underneath the RPMI solution containing the tumor solution.<br><br>For T cell analysis, liver and spleens were homogenized mechanically, filtered through 70 µm nylon mesh, and centrifuged at 400g at 4°C for 15 minutes. Livers underwent density-gradient centrifugation with diluted Percoll (Cytiva, Cat #17089101) to further isolate immune cells. For macrophage analysis, liver and spleen was cut into small pieces and placed for 30 minutes at 37C at 200 RPM in 20mL digestion media containing 4µL of Collagenase IV Stock Solution (Sigma-Aldrich, Cat #C5138) per mL digestion media and 0.5µL of DNAse 1 Stock solution per mL digestion media where digestion was halted using 5% FCS PBS solution on ice. The digested samples were then transferred through 100µM mesh and centrifuged at 50g for 3 minutes. The supernatant was transferred and centrifuged for 400g for 7 minutes. Next, the pellet was resuspended in RPMI and lympholyte was carefully layered underneath this solution. This solution then underwent density-gradient centrifugation with Lympholyte at 850g at room temperature (acceleration 9, deacceleration 0) where the middle layer was kept for further analysis. The red blood cells in the liver and spleen samples were lysed using ACK-lysis buffer, filtered through mesh, centrifuged and resuspended in final appropriate volume.<br><br>Surface staining was done according to manufacturer's recommendation (30-minute incubation time at 4°C.)<br><br>After surface staining, cells were fixed as appropriate using the BD Cytofix/Cytoperm™ Fixation/Permeabilization Kit (BD Biosciences, Cat #554714) or the BD Pharmingen™ Transcription Factor Buffer Set (BD Biosciences, Cat #562574) following the manufacturer's recommendation.
Instrument	Cytoflex LX (376) BA31045 was used for flow analysis.
Software	FlowJo vs 10.10.0 was used to analyze all flow data.
Cell population abundance	Supplemental figure 8 shows purity after isolation of macrophages.
Gating strategy	Gating strategy of T cells is in Supplemental Figure 2 and for macrophages is in Supplemental Figure 7.

☐ Tick this box to confirm that a figure exemplifying the gating strategy is provided in the Supplementary Information.
